# *ABCA1*-Mediated EMT Promotes Papillary Thyroid Cancer Malignancy through the ERK/Fra-1/ZEB1 Pathway

**DOI:** 10.3390/cells12020274

**Published:** 2023-01-10

**Authors:** Ji-Hye Park, Jae-Kyung Myung, Sun-Joo Lee, Hyewon Kim, Soyeon Kim, Seung-Bum Lee, Hyosun Jang, Won-Il Jang, Sunhoo Park, Hyunwon Yang, Sehwan Shim, Min-Jung Kim

**Affiliations:** 1 Laboratory of Radiation Exposure & Therapeutics, National Radiation Emergency Medical Center, Korea Institute of Radiological & Medical Science, Seoul 01812, Republic of Korea; 2 OPTOLANE Technologies Inc., Seongnam 13494, Republic of Korea; 3 Department of Pathology, College of Medicine, Hanyang University, Seoul 01812, Republic of Korea; 4 Laboratory of Experimental Pathology, Departments of Pathology, Korea Institute of Radiological & Medical Science, Seoul 01812, Republic of Korea; 5 Biohealth Convergence, Seoul Women’s University, Seoul 01812, Republic of Korea

**Keywords:** papillary thyroid carcinoma, distant metastasis, ABCA1, epithelial–mesenchymal transition, oncogene

## Abstract

Papillary thyroid cancer (PTC) is the most prevalent histological type of thyroid cancer (TC) worldwide. Although tumor metastasis occurs in regional lymph nodes, distant metastasis (DM) may also occur. Radioactive iodine (RAI) therapy is an effective treatment for TC; however, resistance to RAI occurs in patients with DM. Therefore, in this study, we investigated the efficacy of DM-related biomarkers as therapeutic targets for PTC therapy. ABCA1 expression was higher in aggressive BCPAP cells than in other PTC cells in terms of migration and invasion capacity. The knockdown of *ABCA1* substantially decreased the expression of the epithelial–mesenchymal transition (EMT) marker, N-cadherin, and EMT regulator (ZEB1), resulting in suppressed migration and invasion of BCPAP cells. *ABCA1* knockdown also reduced ERK activity and *Fra-1* expression, which correlated with the effects of an ERK inhibitor or siRNA-mediated inhibition of ERK or Fra-1 expression. Furthermore, *ABCA1*-knocked-down BCPAP cells suppressed cell migration and invasion by reducing Fra-1 recruitment to Zeb1 promoter; lung metastasis was not observed in mice injected with *ABCA1*-knocked-down cells. Overall, our findings suggest that *ABCA1* regulates lung metastasis in TC cells.

## 1. Introduction

Thyroid cancer (TC) is the most common endocrine malignancy. Its incidence in the United States increases by 3.6% annually, with more than three-fold that between 1975 and 2013 [1,2,3,4]. Papillary thyroid carcinoma (PTC), which is the most prevalent thyroid neoplasm affecting 87% of patients with TC, is the least aggressive histological type and has the best overall prognosis [3,5]. The main route of metastasis for PTC is local metastasis to regional lymph nodes (LN) in the neck [6]; however, distant metastasis (DM) sporadically occurs in approximately 5% of patients, most commonly in the lungs and bones [7]. The prognosis of these patients is poor, which is the main cause of TC-related deaths [8]. During the past 70 years, RAI therapy has been a routinely recommended treatment strategy for patients with TC with lung metastasis [9]. Although these treatments usually have a good prognosis, some tumors are resistant to RAI therapy and require alternative treatments [10]. Over the past decade, our understanding of the molecular mechanisms of thyroid carcinogenesis has enabled the development of many new therapeutic agents, such as FDA-approved tyrosine kinase inhibitors and small-molecule inhibitors of VEGFR, BRAF, MEK, NTRK, and RET, which have markedly changed the treatment and outlook of the disease [11].

The ATP-binding cassette (ABC) family of transmembrane proteins is responsible for the transfer of various substrates through extracellular and intracellular membranes [12]. The drug resistance role of ABC transporters has recently been widely considered in cancer cell biology [13]; therefore, the role of these proteins in tumorigenesis is becoming increasingly evident [14]. The regulation of ABC transporter expression and activities of transcription factors or oncogenic signaling pathways, such as PI3K/AKT and MAPK/ERK, plays an important role in tumor initiation and progression [15,16,17]. ABC transporter A1 (ABCA1) is an ABC subfamily A exporter that plays an important role in regulating cellular cholesterol and phospholipid efflux and maintaining lipid homeostasis by participating in the reverse cholesterol transport pathway (RCT) [12]. ABCA1 is closely associated with the development of various human cancers, including thyroid, pancreatic, ovarian, and breast cancers [18,19,20,21]. Nonetheless, its effects on the progression of certain types of cancers remain controversial. Moreover, an association between ABCA1 and metastasis has been recently proposed [22]; however, its underlying molecular mechanisms and roles in metastatic TC remain unelucidated.

In this study, we demonstrated that ABCA1 is highly expressed in patients with PTC with lung metastasis. Moreover, we revealed that the loss of ABCA1 controls the expression of epithelial–mesenchymal transition (EMT) markers via the ERK pathway, consequently leading to reduced lung metastasis in a mouse model. Therefore, we propose that ABCA1 may serve as a metastatic indicator of TC with lung metastasis.

## 2. Materials and Methods

### 2.1. Patients and Surgical Specimens

PTC tissues with lung metastases collected from the Korean Cancer Center Hospital (KCCH, Seoul, Republic of Korea) from 2000 to 2018 were used in this study. Tissues from patients with PTC with lung metastases after lobectomy or complete thyroidectomy were selected based on the pathology reports and medical records. The nine selected primary lesions showed the classical type of PTC on histological examination, and five patients underwent metastasectomy once or twice. Four patients diagnosed with PTC showed no metastasis to the lungs during more than 10 years of follow-up.

### 2.2. Cell Culture and Chemical Reagents

Normal human primary thyroid follicular epithelial cells, Nthy-ori 3-1 (Nthy), were purchased from Sigma (St. Louis, MO, USA). The papillary thyroid carcinoma cell lines, BHP10-3, BCPAP, and SNU790, were purchased from the Korean Cell Line Bank (Seoul, Republic of Korea). The cells were cultured in Roswell Park Memorial Institute (RPMI) 1640 medium (Invitrogen, Carlsbad, CA, USA) supplemented with 10% fetal bovine serum and antibiotics in a 37 °C incubator with 5% CO_2_. For transient silencing of the *ABCA1*, *ERK*, *ZEB1*, and *Fra-1* genes, cells were transfected with non-targeting siRNA and siRNAs against the target genes, as mentioned in Table S1. The siRNAs were purchased from Integrated DNA Technologies (San Diego, CA, USA) and transfected into cells for 48 h using Lipofectamine 2000 (Invitrogen), as described by the manufacturer. U0126, an ERK inhibitor, was purchased from Calbiochem (San Diego, CA, USA).

### 2.3. Digital Real-Time PCR

Digital real-time PCR (Dr. PCR) was performed on the LOAA (Optolane, Seongnam, Republic of Korea) for quantifying *ABCA1* expression in patients with TC with or without lung metastasis, using a Taqman probe. In brief, a total of 30 μL reaction mixture contained 15 μL 2X Dr. PCR Master mix (Optolane, Seongnam, Republic of Korea) and 7.5 μL ABCA1 (FAM)/GAPDH (FRET) duplex assays (20 pmol forward primer, 20 pmol reverse primer, 4 pmol FAM probe, and 4 pmol FRET probe per reaction) was loaded into the cartridge, and PCR was performed with LOAA (Optolane). The reactions for ABCA1/GAPDH duplex assay were conducted under the conditions of 3 min at 50 °C, 15 min at 95 °C, and 50 cycles of 10 s at 95 °C and 30 s at 60 °C. After amplification, the results were analyzed using Dr. PCR Analyzer (Optolane). The specific primers and probes used are provided in Table S2.

### 2.4. Transwell Migration and Invasion Assays

Cell migration and invasion were evaluated as previously described [23]. Briefly, for the migration assay, cells were seeded in the upper chamber of a transwell assay (Corning Inc., Corning, NY, USA) and incubated for 24 h. For the invasion assay, cells were plated in the upper compartments of the growth-reduced Matrigel-coated chambers (BD Biosciences, Franklin Lakes, NJ, USA) for 48 h. To observe the cells that migrated to the lower chamber, the transwell membranes were fixed with 4% PFA and stained with 0.05% crystal violet (Sigma, St. Louis, MO, USA). Cells on the undersurface of the membrane were counted under a light microscope (Olympus, Tokyo, Japan).

### 2.5. Wound-Healing Assay

A wound-healing assay was performed to analyze the changes in cell motility by regulating *ABCA1* expression. After culturing the cells to 80% confluence, the cell monolayers were scratched with a 200 μL pipette tip, following which both cell lines were further incubated with fresh medium without treatment for 24 h. Photomicrographs were then taken at 100× magnification using an inverted microscope (Olympus, Tokyo, Japan).

### 2.6. Statistical Analysis

The statistical significance of the differences between mean values was calculated via unpaired Student’s *t*-tests using SPSS (version 12.0; SPSS Inc., Chicago, IL, USA) or Excel (Microsoft, Redmond, WA, USA). Statistical significance was set at *p* < 0.05.

## 3. Results

### 3.1. Characterization of PTC Cell Line for a Mouse Lung Metastasis Model

**Figure 1:**
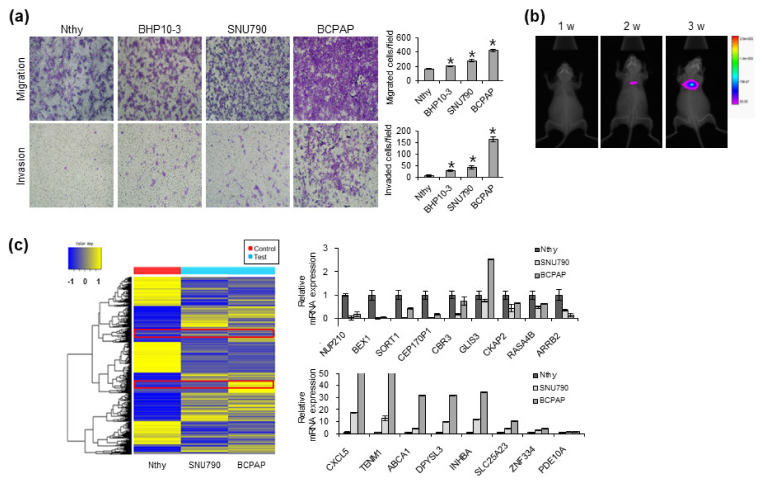
Characterization of papillary thyroid cell lines for metastatic capacity. (**a**) Migration (upper) and invasion (lower) by thyroid cells were analyzed and quantified. * *p* < 0.05 compared with Nthy cells (Student’s *t*-test). (**b**) Images of lung metastases that developed in the BCPAP cells in the tail vein injection models. The images were acquired using an IVIS imaging system. Representative luciferase signals captured in each group at the time of the initial injection, 1 week, 2 weeks, and 3 weeks after cell injection, are shown. (**c**) Heatmap generated from the microarray analysis of indicated thyroid cells showing the differential expression between the three cells. The mRNA levels of indicated candidate targets in the thyroid cell lines were validated via qRT-PCR. The data represent the mean ± SD of triplicate measurements. * *p* < 0.05 vs. Nthy cells based on two-tailed Student’s *t*-test.

Approximately 30% of patients with TC developed metastasis. Although most of these metastases were limited to the regional LN of the neck, 1–4% of cases showed DM to other organs, such as the lungs and bones [7]. Because metastasis is characterized by cell migration and invasion abilities, we first investigated the cell migration ability of various PTC cells. By screening the migratory and invasive capacities of several thyroid immortalized and cancer cells, we found that BCPAP cells had the best cell migration and invasion capacities (Figure 1a). We first established BCPAP luciferase-stable cells for visualization, and a tail vein injection model was established in nude mice. Lung metastasis experiments revealed that BCPAP cells metastasized to the lungs (Figure 1b). To profile the expression patterns of genes that regulate lung metastasis of PTC, we used microarray technology to compare thyroid cell lines with different metastatic potentials. The expressions of several genes were substantially altered in BCPAP cells than in Nthy and SNU790 cells. To confirm our microarray results, we examined the mRNA expression levels of candidate genes (Figure 1c). We observed increased expression of ABCA1 in BCPAP cells because ABCA1 is known to be associated with a malignant phenotype in several types of cancers [24,25,26,27]. Epidemiological and experimental evidence suggests that ABCA1 may be involved in the progression of certain cancer types [4]. Therefore, these results suggest that it is involved in lung metastasis of PTC.

### 3.2. ABCA1 Expression in Various PTC Cell Lines and Patients with Cancer with Metastatic Ability

**Figure 2:**
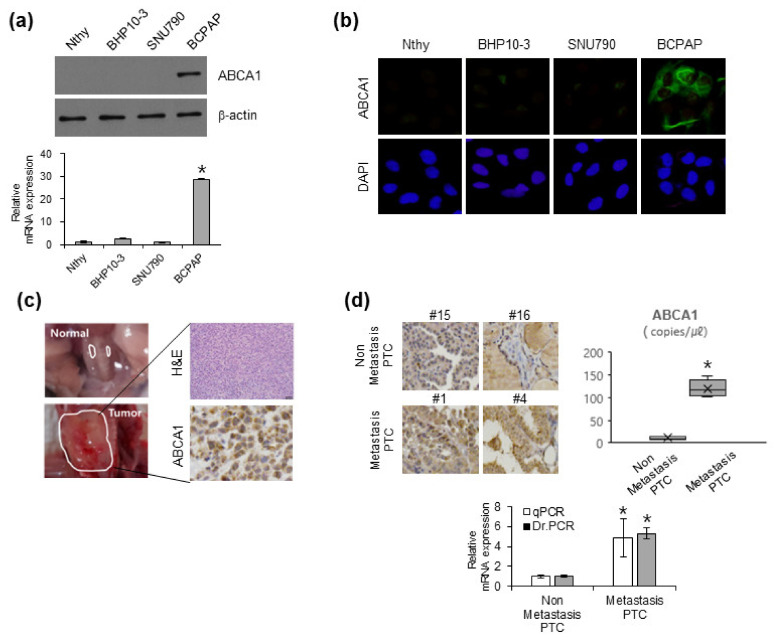
ABCA1 expression in papillary thyroid cancer cells and patients suffering from cancer with lung metastasis. (**a**,**b**) Expression levels of ABCA1 in Nthy immortalized non-cancer cells and three thyroid cancer cell lines were analyzed via Western blotting, qRT-PCR, and immunocytochemistry. DAPI was used to stain the nuclei of cells. (**c**) Representative H&E and ABCA1-stained tumor tissues from mice injected with BCPAP cells. Scale bar: 100 μm. Data are presented as the mean ± SEM (n = 4, independent experiments). (**d**) Representative IHC images showing ABCA1 levels in a sample from a patient with thyroid cancer with lung metastasis (upper left). Scale bar: 100 μm. Differences in the expression levels of ABCA1 in FFPE of patients with thyroid cancer with or without lung metastases were analyzed via qRT-PCR (lower) and Dr. PCR (upper right and lower). * *p* < 0.05 (Fisher’s exact test).

Many studies have revealed that ABCA1 is associated with a malignant phenotype in various carcinomas, including breast, colon, and lung cancers and melanoma [24,25,26,27]. To clarify the role of ABCA1 in lung metastasis of PTC, we investigated its expression in several immortalized thyroid and cancer cells. ABCA1 expression was the highest in BCPAP cells among various thyroid cell lines (Figure 2a,b). To confirm whether ABCA1 was consistent with the in vivo and in vitro results of BCPAP cells, we performed orthotopic xenografts in BALB/c nude mice. Mice injected with BCPAP cells showed an increase in tumor volume compared with the mice in the control group. As the SNU790 cell line did not form a tumor mass [28], we used the BCPAP cell line. ABCA1 was highly expressed in tumors generated from orthotopic xenografted mice with BCPAP cells, which showed metastatic ability in the lungs (Figure 1b and Figure 2c). To investigate the possibility of using digital real-time PCR (Dr. PCR) for ABCA1 evaluation in FFPE tissues, we evaluated the probe-based absolute expression of ABCA1 in patients with PTC with and without lung metastases. In FFPE tissues of PTC with and without lung metastases, the copy numbers of ABCA1 per μL were 11.9 ± 2.68 and 126.25 ± 17.92, respectively (*p* < 0.01) (Figure 2d, upper right). The relative fold change in ABCA1 expression normalized to the reference gene showed less data deviation in Dr. PCR analysis than in the qPCR analysis. Consequently, PTC tissues from patients with lung metastasis showed higher expression of ABCA1 than those of patients without metastasis (Figure 2d). Overall, these results suggest that ABCA1 contributes to lung metastasis both in vitro and in vivo.

### 3.3. ABCA1 Expression Leads to EMT Conversion, Motility, and Invasion by Targeting EMT-TF

**Figure 3:**
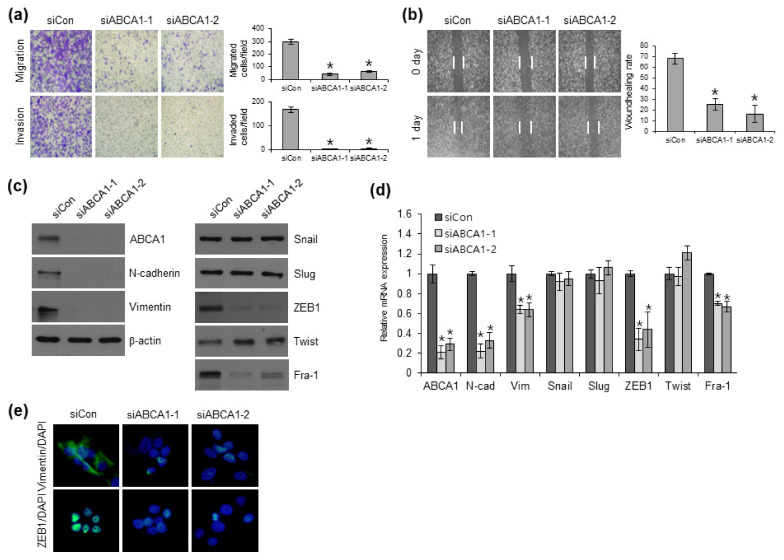
siRNA-mediated ABCA1 knockdown inhibits migration and invasion by targeting epithelial–mesenchymal transition transcription factors (EMT-TF). (**a**) Analysis of migration (upper) and invasion (lower) in ABCA1-knocked-down BCPAP cells. * *p* < 0.05 versus siCon (Student’s *t*-test). (**b**) Wound-healing capacity after ABCA1 knockdown was examined in BCPAP cells. After 24 h, five different wounds in the picture were randomly selected and measured for quantification. (**c**–**e**) Expression levels of the indicated EMT-related proteins in siRNA-mediated ABCA1-knocked-down BCPAP cells were analyzed via Western blotting, qRT-PCR, and immunocytochemistry. DAPI was used to stain the nuclei of cells. The data in (**a**,**d**) represent the means ± S.D. of triplicate assays.

To investigate the effects of ABCA1 on EMT progression, we examined whether ABCA1 affects the migration and invasion of BCPAP cells. siRNA-mediated ABCA1 knockdown in BCPAP cells decreased their migration and invasion in BCPAP cells (Figure 3a). ABCA1-knocked-down BCPAP cells transfected with siRNAs also showed decreased wound-healing activity (Figure 3b). Transient ABCA1 knockdown in BCPAP cells using ABCA1 siRNAs led to the downregulation of mesenchymal markers (N-cadherin and vimentin) and EMT regulators (ZEB1 and Fra-1) at the protein and mRNA levels (Figure 3c,d). Consistent with the results of Western blotting and qRT-PCR, the immunocytochemistry (ICC) results also showed decreased expression of vimentin and ZEB1 in siRNA-mediated ABCA1-knocked-down BCPAP cells (Figure 3e). Collectively, these results indicated that ABCA1 plays an important role in EMT in PTC by regulating the expression of EMT-related molecules.

### 3.4. ABCA1 Regulates EMT through the ERK Pathway

**Figure 4:**
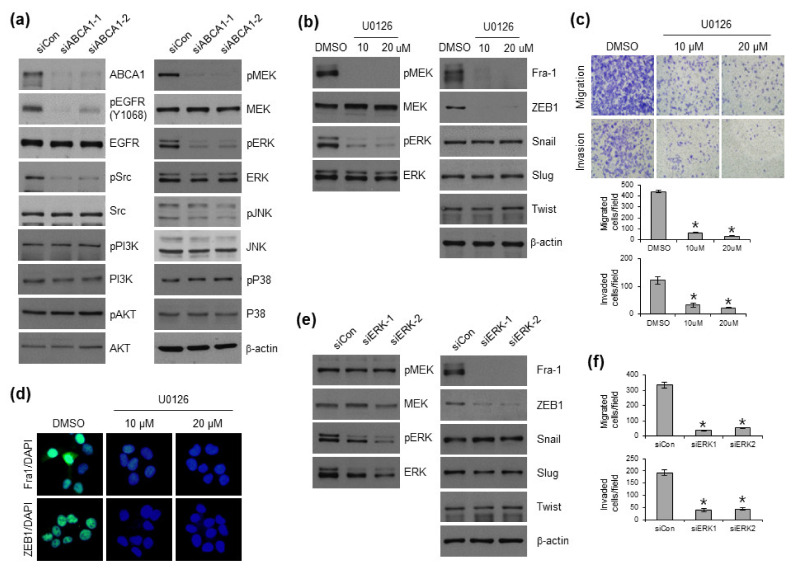
Knockdown of ABCA1 using siRNAs suppresses EMT by inhibiting the ERK pathway. (**a**) Protein expressions of the indicated signaling pathways in siRNA-mediated ABCA1-knocked-down BCPAP cells were measured using Western blotting. Expression levels of the indicated proteins in BCPAP cells were analyzed after treatment with U0126 (**b**) or siRNA against ERK (**e**) via Western blotting. (**c**) Migration (upper) and invasion (lower) of U0126-treated BCPAP cells were analyzed and quantified. * *p* < 0.05, compared to DMSO-treated cells (Student’s *t*-test). (**d**) Effects of U0126 on Fra-1 and ZEB1 expressions were examined via immunofluorescence staining. (**f**) Graphs show the migration (upper) and invasion (lower) abilities of siRNA-mediated ERK-knocked-down BCPAP cells.

To determine whether the EMT regulatory signaling pathway is dependent on ABCA1 expression, we identified the most aberrantly activated pathways, including the EGFR, SRC, and ERK pathways, which are associated with ABCA1 in various carcinomas [29,30]. Moreover, Fra-1 is a critical EMT effector downstream of Ras signaling that regulates EMT by regulating ZEB1 [31,32,33]. Therefore, we also examined the involvement of Ras downstream of the MAPK signaling pathway in the regulation of EMT by ABCA1. According to the data, phosphorylated MEK and ERK were markedly decreased in response to ABCA1 expression, indicating that ABCA1 may regulate EMT through the MEK/ERK pathway (Figure 4a). To further clarify the role of ERK in EMT progression in TC cells, we elucidated the molecular mechanism underlying the regulation of EMT-TF expression by ERK activity. Based on a previous finding that MEK/ERK inhibitor induced Fra-1 expression [34], we confirmed that the MEK/ERK inhibitor, U0126, regulates not only Fra-1 protein expression but also ZEB1 (Figure 4b). In addition, BCPAP cells treated with U0126 showed decreased motility and invasiveness in the transwell migration and invasion assays (Figure 4c). ICC results also showed decreased expression of Fra-1 and ZEB1 in BCPAP cells treated with U0126 (Figure 4d). Furthermore, transient ERK knockdown in BCPAP cells using two different siRNAs led to the downregulation of Fra-1 and ZEB1 and inhibition of migration and invasion abilities (Figure 4e,f). Overall, these data show that ERK is involved in EMT in BCPAP cells and regulates Fra-1 and ZEB1.

### 3.5. Knockdown of ABCA1 Expression Suppresses ZEB1 Expression via ERK/Fra1

**Figure 5:**
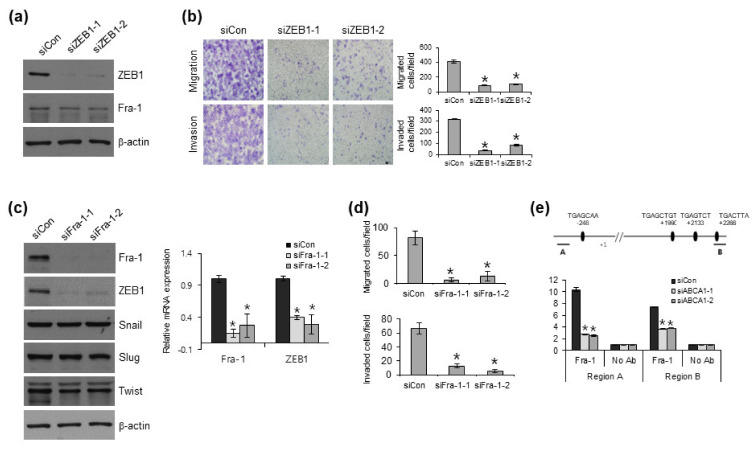
Knockdown of ABCA1 expression suppresses ZEB1 expression via ERK/Fra-1. (**a**) Effect of ZEB1 on Fra-1 expression was analyzed via Western blotting. (**b**) Representative images and quantification of migration (upper) and invasion (lower) by the indicated cells. The data represent the means ± S.D. of triplicate assays. * *p* < 0.05, compared with siCon (Student’s *t*-test). (**c**) Effect of Fra-1 on ZEB1 expression was analyzed via Western blotting (left) and qRT-PCR (right). (**d**) Graphs show the migration (upper) and invasion (lower) abilities of siRNA-mediated Fra-1-knocked-down BCPAP cells. (**e**) ChIP analysis showing the recruitment of Fra-1 to the promoter regions of *ZEB1* gene in ABCA1-knocked-down BCPAP cells. Results are shown as means ± S.D. of experiments in triplicate. * *p* < 0.05 vs. siCon (Student’s *t*-test).

Because ZEB1 expression can be regulated by ERK [8] and ERK/Fra-1 regulates genes related to cell migration, we investigated whether Fra-1 may be involved in the regulation of ZEB1 via the ERK pathway. To determine the relationship between Fra-1 and ZEB1, we first examined the effect of ZEB1 on Fra-1 expression in BCPAP cells. siRNA-mediated ZEB1 knockdown did not change Fra-1 protein expression but reduced cell migration and invasion in BCPAP cells (Figure 5a,b). In contrast, siRNA-mediated inhibition of Fra-1 expression suppressed both the protein and mRNA expression levels of ZEB1 and reduced cell migration and invasion in BCPAP cells (Figure 5c–e). As ZEB1 expression can be regulated by ERK/Fra-1 [35,36], we hypothesized that ABCA1 may be involved in the regulation of ZEB1 via the ERK/Fra-1 pathway. Consistent with a previous study showing the enrichment of Fra-1 in the ZEB1 promoter [36], ABCA1-knocked-down BCPAP cells reduced Fra-1 recruitment to the ZEB1 promoter (Figure 5e). Collectively, these data support the notion that ABCA1 induces EMT gene expression programs, including Fra1 and ZEB1, via the ERK pathway in BCPAP cells, which is characteristic of EMT and invasiveness.

### 3.6. ABCA1 Is Associated with Lung Metastasis of Papillary Thyroid Cancer Cells

**Figure 6:**
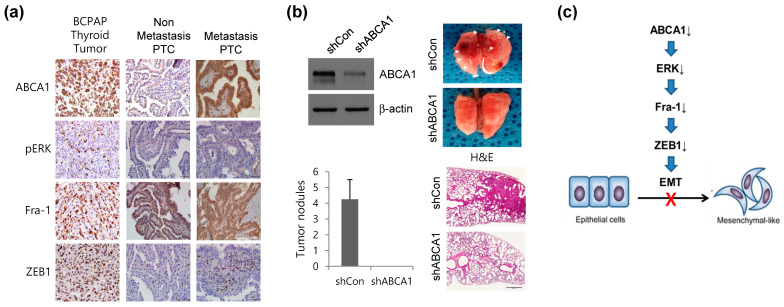
ABCA1 is associated with lung metastasis. (**a**) Representative images indicating protein-stained tumor tissues from mice injected with BCPAP cells and patients with thyroid cancer. Scale bar: 100 μm. (**b**) Cell lysates from BCPAP cells stably expressing control short hairpin RNA (shRNA: shCon) or ABCA1 shRNA (shABCA1) were analyzed via immunoblotting (upper). A representative image of histological analysis of the lungs isolated from mice injected with shCon in the tail vein or ABCA1-knocked-down (shABCA1) BCPAP cells (left). Arrowheads and hematoxylin and eosin (H&E) staining images indicate lung metastatic nodules (right). Data were quantified by counting the number of surface lung nodules (upper left). Error bars indicate the means ± S.E.M. * *p* < 0.05 versus shCon (Student’s *t*-test). (**c**) Proposed model for the regulation of EMT by ABCA1. In the absence of ABCA1, decreased expression of Fra-1 is due to reduced ERK activity, which leads to decreased Fra-1 binding to the promoter region of *ZEB1* EMT-TF. This reduces the expression of ZEB1, and the reduced expression of ZEB1 suppresses EMT.

To determine whether ABCA1 is involved in inducing lung metastasis, we confirmed the expression of ABCA1 and target proteins, such as pERK, Fra-1, and ZEB1, in BCPAP cell-bearing PTC. Consistent with our in vitro results, ABCA1 expression was positively correlated with pERK/Fra-1/ZEB1 expression in mice bearing orthotopic xenograft BCPAP cells (Figure 6a, left panel). Moreover, the expression of these proteins in the tissues of patients with PTC with lung metastases was higher than that in the tissues of patients without lung metastases (Figure 6a, right panel). Mice injected with stable ABCA1-knocked-down BCPAP cells showed a marked reduction in lung metastasis compared with mice injected with BCPAP cells (Figure 6b, left panel). Four weeks after lung resection, macroscopically visible metastases were detected in mice injected with BCPAP; however, mice injected with ABCA1-knocked-down BCPAP cells showed no visible metastases (Figure 6b, right upper panel). Hematoxylin and eosin (H&E) staining also revealed the presence of metastatic nodules in the resected lungs of mice injected with BCPAP cells; however, no metastases were detected in the lungs of mice injected with ABCA1-knocked-down cells (Figure 6b, right bottom panel). Collectively, we concluded that ABCA1 loss inhibited Fra-1 and ZEB1 through ERK phosphorylation inhibition and subsequently suppressed EMT, thereby suppressing lung metastasis (Figure 6c).

## 4. Discussion

In this study, we investigated the efficacy of DM-related biomarkers as therapeutic targets for PTC therapy and demonstrated that *ABCA1* plays an important role in the inhibition of lung metastasis of PTC via the ERK/Fra-1/ZEB1 pathway. *ABCA1* is highly expressed not only in BCPAP cells with high cell invasion and mobility but also in BCPAP-bearing tumor tissues. In addition, *ABCA1* expression is high in the tissues of patients with PTC with lung metastases. siRNA-mediated *ABCA1*-knocked-down BCPAP cells showed reduced cell invasion, migration, and expression of EMT regulators. These events were similar to the effects of inhibition of ERK, Fra-1, and ZEB1 by specific inhibitors or siRNAs, consequently leading to EMT inhibition. Therefore, we propose that the ERK/Fra-1/ZEB1-dependent EMT cascade is controlled by *ABCA1*, thereby suppressing lung metastasis as an EMT modulator.

Our findings demonstrated that *ABCA1* may play an important role in EMT in PTC cells. As the role of *ABCA1* in tumorigenesis has become increasingly evident, there is increasing evidence that *ABCA1* contributes to cancer development as well as malignant phenotypes and drug resistance [13,14,22]. Because cancer cells are closely related to intracellular cholesterol levels and *ABCA1* functions as a reverse cholesterol transport channel [12,37,38], the role of *ABCA1* in the regulation of cellular processes involved in tumor behavior remains controversial. Some studies have revealed that *ABCA1* may affect cancer cell growth and migration by regulating the cholesterol levels [39,40,41,42]. Decreased *ABCA1* expression in several cancers, such as prostate, breast, and oral cancers, is associated with the rate of cancer cell proliferation [43,44,45,46], and ABCA1 overexpression substantially inhibits the proliferation, migration, and invasion of lung adenocarcinoma cells [47,48]. Similarly, *ABCA1* hypermethylation may be associated with enhanced cell proliferation and inhibition of apoptosis [16,49,50]. Evidence from the past decade has suggested that body fat percentage, cholesterol levels, and obesity may be associated with a PTC type [51,52]. Moreover, although a recent study revealed an association between cholesterol and malignancy in thyroid cancer [18], the relationship between *ABCA1* and TC remains unknown. Further studies are required to establish the role of *ABCA1* in TC and cholesterol metabolism. Nevertheless, our findings provide evidence that cooperation between the *ABCA1* and ERK pathways is an important molecular connection in EMT. A recent study suggested that potential inhibition of ERK signaling is required to properly induce iodide uptake as a promising strategy for the treatment of TC [53]. Consistent with this evidence, we showed that *ABCA1* regulates the transcription of *ZEB1*, an EMT-TF, by regulating the ERK/Fra-1 pathway. Furthermore, our novel findings showed that ABCA1 decreased *Fra-1* expression and then inhibited Fra-1-mediated *ZEB1* transcription, similar to the ERK inhibitory effect. Overall, we propose that *ABCA1* may have an oncogenic function as a novel regulator of ERK and Fra-1.

Our data emphasize the potential of *ABCA1* expression as a predictive marker of lung metastasis in patients with PTC. Accurate nucleic acid measurement of specific markers is receiving great attention not only for cancer diagnosis and prognosis but also for tracking the evolution of cancer cells before and after treatment. Digital PCR has recently emerged as a promising technology for gene quantitative research and diagnosis because it can quantify the expression level of a target gene in 20,000 independent partitions, ensuring high specificity and sensitivity because it is less affected by various PCR inhibitors [54,55,56]. Moreover, samples embedded for long periods are susceptible to non-specific binding of SYBR in RT-PCR analysis because they have a negative effect on the quality of DNA and RNA that can be isolated from FFPE tissues. To confirm the potential of *ABCA1* as a metastasis predictive marker, we applied our novel Dr. PCR method to analyze tumors with and without lung metastases, previously analyzed via qPCR. Some studies have revealed that high *ABCA1* expression promotes migration and growth and reduces clinical outcomes [22,51,57]. Similarly, Pan et al. suggested that ABCA1 is a specific marker of triple-negative breast cancer (TNBC), as its expression is higher in TNBC tissues than in non-cancerous mammary tissues [27]. A recent study revealed that *ABCA1* was upregulated in the most aggressive samples, although these changes were not substantial in advanced thyroid cancers, including poorly differentiated TC and anaplastic TC [18]. Consistent with this, our in vitro and in vivo data showed an association between high *ABCA1* expression and the invasion and metastasis of human PTC, supporting the clinical significance of *ABCA1* in PTC with lung metastasis. In summary, we demonstrated that *ABCA1* functions as a modulator of EMT via the ERK/Fra-1/ZEB1 pathway and is a novel predictor of response to TC with lung metastasis. Therefore, *ABCA1* may be an important oncogene and novel target in TC with lung metastasis.

## Data Availability

Not applicable.

## Supplementary Materials

The following supporting information can be downloaded at: https://www.mdpi.com/article/10.3390/cells12020274/s1, Table S1: siRNA sequences used in this study; Table S2: Primers and probes of Dr. PCR used in this study; Table S3: Primers of qRT-PCR used in this study; Table S4: Primers of ChIP-qPCR used in this study. Reference [58] is cited in the Supplementary Materials.

## References

[1] Jemal A., Ward E.M., Johnson C.J., Cronin K.A., Ma J., Ryerson A.B., Mariotto A., Lake A.J., Wilson R., Sherman R.L. (2017). Annual report to the nation on the status of cancer, 1975–2014, featuring survival. J. Natl. Cancer Inst..

[2] Shobab L., Gomes-Lima C., Zeymo A., Feldman R., Jonklaas J., Wartofsky L., Burman K.D. (2019). Clinical, pathological, and molecular profiling of radioactive iodine refractory differentiated thyroid cancer. Thyroid.

[3] Lim H., Devesa S., Sosa J., Check D., Kitahara C. (2017). Trends in Thyroid Cancer Incidence and Mortality in the United States, 1974–2013. JAMA.

[4] Sherma S.I.J.T.L. (2003). Thyroid Carcinoma. Lancet.

[5] Yan K.L., Li S., Tseng C.H., Kim J., Nguyen D.T., Dawood N.B., Livhits M.J., Yeh M.W., Leung A.M. (2020). Rising incidence and incidence-based mortality of thyroid cancer in California, 2000–2017. J. Clin. Endocrinol. Metab..

[6] Erden E.S., Babayigit C., Davran R., Akin M., Karazincir S., Isaogullari N., Demirkose M., Genc S. (2013). Papillary thyroid carcinoma with lung metastasis arising from dyshormonogenetic goiter: A case report. Case Rep. Med..

[7] Borschitz T., Eichhorn W., Fottner C., Hansen T., Schad A., Schadmand-Fischer S., Weber M.M., Schreckenberger M., Lang H., Musholt T.J. (2010). Diagnosis and treatment of pancreatic metastases of a papillary thyroid carcinoma. Thyroid.

[8] Nakanishi K., Kikumori T., Miyajima N., Takano Y., Noda S., Takeuchi D., Iwano S., Kodera Y. (2018). Impact of patient age and histological type on radioactive iodine avidity of recurrent lesions of differentiated thyroid carcinoma. Clin. Nucl. Med..

[9] Qiu Z.L., Shen C.T., Sun Z.K., Song H.J., Zhang G.Q., Luo Q.Y. (2019). Lung metastases from papillary thyroid cancer with persistently negative thyroglobulin and elevated thyroglobulin antibody levels during radioactive iodine treatment and follow-up: Long-term outcomes and prognostic indicators. Front. Endocrinol..

[10] Kaae A.C., Kreissl M.C., Krüger M., Infanger M., Grimm D., Wehland M. (2021). Kinase-inhibitors in iodine-refractory differentiated thyroid cancer-focus on occurrence, mechanisms, and management of treatment-related hypertension. Int. J. Mol. Sci..

[11] Fullmer T., Cabanillas M.E., Zafereo M. (2021). Novel therapeutics in radioactive iodine-resistant thyroid cancer. Front. Endocrinol..

[12] Xiong T., Xu G., Huang X.L., Lu K.Q., Xie W.Q., Yin K., Tu J. (2018). ATP-binding cassette transporter A1: A promising therapy target for prostate cancer. Mol. Clin. Oncol..

[13] Nobili S., Lapucci A., Landini I., Coronnello M., Roviello G., Mini E. (2020). Role of ATP-binding cassette transporters in cancer initiation and progression. Semin. Cancer Biol..

[14] Muriithi W., Macharia L.W., Heming C.P., Echevarria J.L., Nyachieo A., Filho P.N., Neto V.M. (2020). ABC transporters and the hallmarks of cancer: Roles in cancer aggressiveness beyond multidrug resistance. Cancer Biol. Med..

[15] Fletcher J.I., Haber M., Henderson M.J., Norris M.D. (2010). ABC transporters in cancer: More than just drug efflux pumps. Nat. Rev. Cancer.

[16] Chou J.L., Huang R.L., Shay J., Chen L.Y., Lin S.J., Yan P.S., Chao W.T., Lai Y.H., Lai Y.L., Chao T.K. (2015). Hypermethylation of the TGF-beta target, ABCA1 is associated with poor prognosis in ovarian cancer patients. Clin. Epigenet..

[17] Crawford R.R., Potukuchi P.K., Schuetz E.G., Schuetz J.D. (2018). Beyond competitive inhibition: Regulation of ABC transporters by kinases and protein-protein interactions as potential mechanisms of drug-drug interactions. Drug Metab. Dispos..

[18] Revilla G., Pons M.P., Baila-Rueda L., García-León A., Santos D., Cenarro A., Magalhaes M., Blanco R.M., Moral A., Ignacio Pérez J. (2019). Cholesterol and 27-hydroxycholesterol promote thyroid carcinoma aggressiveness. Sci. Rep..

[19] Mohelnikova-Duchonova B., Brynychova V., Oliverius M., Honsova E., Kala Z., Muckova K., Soucek P. (2013). Differences in transcript levels of ABC transporters between pancreatic adenocarcinoma and nonneoplastic tissues. Pancreas.

[20] Chien J., Fan J.B., Bell D.A., April C., Klotzle B., Ota T., Lingle W.L., Gonzalez Bosquet J., Shridhar V., Hartmann L.C. (2009). Analysis of gene expression in stage I serous tumors identifies critical pathways altered in ovarian cancer. Gynecol. Oncol..

[21] Park S., Shimizu C., Shimoyama T., Takeda M., Ando M., Kohno T., Katsumata N., Kang Y.K., Nishio K., Fujiwara Y. (2006). Gene expression profiling of ATP-binding cassette (ABC) transporters as a predictor of the pathologic response to neoadjuvant chemotherapy in breast cancer patients. Breast Cancer Res. Treat..

[22] Zhao W., Prijic S., Urban B.C., Tisza M.J., Zuo Y., Li L., Tan Z., Chen X., Mani S.A., Chang J.T. (2016). Candidate antimetastasis drugs suppress the metastatic capacity of breast cancer cells by reducing membrane fluidity. Cancer Res..

[23] Park J.H., Kim Y.H., Park E.H., Lee S.J., Kim H., Kim A., Lee S.B., Shim S., Jang H., Myung J.K. (2019). Effects of metformin and phenformin on apoptosis and epithelial-mesenchymal transition in chemoresistant rectal cancer. Cancer Sci..

[24] Bachmeier B.E., Iancu C.M., Killian P.H., Kronski E., Mirisola V., Angelini G., Jochum M., Nerlich A.G., Pfeffer U. (2009). Overexpression of the ATP binding cassette gene ABCA1 determines resistance to curcumin in M14 melanoma cells. Mol. Cancer.

[25] Prochazka L., Koudelka S., Dong L.F., Stursa J., Goodwin J., Neca J., Slavik J., Ciganek M., Masek J., Kluckova K. (2013). Mitochondrial targeting overcomes ABCA1-dependent resistance of lung carcinoma to alpha-tocopheryl succinate. Apoptosis.

[26] Dang C.V. (2013). MYC, metabolism, cell growth, and tumorigenesis. Cold Spring Harb. Perspect. Med..

[27] Pan H., Zheng Y., Pan Q., Chen H., Chen F., Wu J., Di D. (2019). Expression of LXR- β, ABCA1 and ABCG1 in human triple-negative breast cancer tissues. Oncol. Rep..

[28] Kim Y.H., Choi Y.W., Lee J., Soh E.Y., Kim J.H., Park T.J. (2017). Senescent tumor cells lead the collective invasion in thyroid cancer. Nat. Commun..

[29] Gabitova L., Restifo D., Gorin A., Manocha K., Handorf E., Yang D.H., Cai K.Q., Klein-Szanto A.J., Cunningham D., Kratz L.E. (2015). Endogenous sterol metabolites regulate growth of EGFR/KRAS-dependent tumors via LXR. Cell Rep..

[30] Fang R., Chen X., Zhang S., Shi H., Ye Y., Shi H., Zou Z., Li P., Guo Q., Ma L. (2021). EGFR/SRC/ERK-stabilized YTHDF2 promotes cholesterol dysregulation and invasive growth of glioblastoma. Nat. Commun..

[31] Cranshaw I.M., Carnaille B. (2008). Micrometastases in thyroid cancer. An important finding?. Surg. Oncol..

[32] Liu L.S., Liang J., Li J.H., Liu X., Jiang L., Long J.X., Jiang Y.M., Wei Z.X. (2017). The incidence and risk factors for central lymph node metastasis in cN0 papillary thyroid microcarcinoma: A meta-analysis. Eur. Arch. Otorhinolaryngol..

[33] Randolph G.W., Duh Q.Y., Heller K.S., LiVolsi V.A., Mandel S.J., Steward D.L., Tufano R.P., Tuttle R.M., American Thyroid Association Surgical Affairs Committee’s Taskforce on Thyroid Cancer Nodal Surgery (2012). The prognostic significance of nodal metastases from papillary thyroid carcinoma can be stratified based on the size and number of metastatic lymph nodes, as well as the presence of extranodal extension. Thyroid.

[34] Li C., Wu Q., Sun S.J.C.C. (2020). Radioactive iodine therapy in patients with thyroid carcinoma with distant metastases: A SEER-based study. Cancer Control..

[35] Shin S., Buel G.R., Wolgamott L., Plas D.R., Asara J.M., Blenis J., Yoon S.O. (2015). ERK2 mediates metabolic stress response to regulate cell fate. Mol. Cell.

[36] Bakiri L., Macho-Maschler S., Custic I., Niemiec J., Guío-Carrión A., Hasenfuss S.C., Eger A., Müller M., Beug H., Wagner E.F. (2015). Fra-1/AP-1 induces EMT in mammary epithelial cells by modulating Zeb1/2 and TGFbeta expression. Cell Death Differ..

[37] Oram J.F., Yokoyama S. (1996). Apolipoprotein-mediated removal of cellular cholesterol and phospholipids. J. Lipid Res..

[38] Ikonen E. (2008). Cellular cholesterol trafficking and compartmentalization. Nat. Rev. Mol. Cell Biol..

[39] Jacobo-Albavera L., Domínguez-Pérez M., Medina-Leyte D.J., González-Garrido A., Villarreal-Molina T. (2021). The role of the ATP-binding cassette A1 (ABCA1) in human disease. Int. J. Mol. Sci..

[40] Tabas I. (2002). Consequences of cellular cholesterol accumulation: Basic concepts and physiological implications. J. Clin. Investig..

[41] Huang B., Song B.L., Xu C. (2020). Cholesterol metabolism in cancer: Mechanisms and therapeutic opportunities. Nat. Metab..

[42] Kitahara C.M., Berrington de González A., Freedman N.D., Huxley R., Mok Y., Jee S.H., Samet J.M. (2011). Total cholesterol and cancer risk in a large prospective study in Korea. J. Clin. Oncol..

[43] Fukuchi J., Hiipakka R.A., Kokontis J.M., Hsu S., Ko A.L., Fitzgerald M.L., Liao S. (2004). Androgenic suppression of ATP-binding cassette transporter A1 expression in LNCaP human prostate cancer cells. Cancer Res..

[44] Schimanski S., Wild P.J., Treeck O., Horn F., Sigruener A., Rudolph C., Blaszyk H., Klinkhammer-Schalke M., Ortmann O., Hartmann A. (2010). Expression of the lipid transporters ABCA3 and ABCA1 is diminished in human breast cancer tissue. Horm. Metab. Res..

[45] Moon S.H., Huang C.H., Houlihan S.L., Regunath K., Freed-Pastor W.A., Morris J.P., Tschaharganeh D.F., Kastenhuber E.R., Barsotti A.M., Culp-Hill R. (2019). p53 represses the mevalonate pathway to mediate tumor suppression. Cell.

[46] Kaneko T., Kanno C., Ichikawa-Tomikawa N., Kashiwagi K., Yaginuma N., Ohkoshi C., Tanaka M., Sugino T., Imura T., Hasegawa H. (2015). Liver X receptor reduces proliferation of human oral cancer cells by promoting cholesterol efflux via up-regulation of ABCA1 expression. Oncotarget.

[47] Liu K., Zhang W., Tan J., Ma J., Zhao J. (2019). MiR-200b-3p functions as an oncogene by targeting ABCA1 in lung adenocarcinoma. Technol. Cancer Res. Treat..

[48] Hedditch E.L., Gao B., Russell A.J., Lu Y., Emmanuel C., Beesley J., Johnatty S.E., Chen X., Harnett P., George J. (2014). ABCA transporter gene expression and poor outcome in epithelial ovarian cancer. J. Natl Cancer Inst..

[49] Lee B.H., Taylor M.G., Robinet P., Smith J.D., Schweitzer J., Sehayek E., Falzarano S.M., Magi-Galluzzi C., Klein E.A., Ting A.H. (2013). Dysregulation of cholesterol homeostasis in human prostate cancer through loss of ABCA1. Cancer Res..

[50] Bi D.P., Yin C.H., Zhang X.Y., Yang N.N., Xu J.Y. (2016). MiR-183 functions as an oncogene by targeting ABCA1 in colon cancer. Oncol. Rep..

[51] Kim H.J., Kim N.K., Choi J.H., Sohn S.Y., Kim S.W., Jin S.M., Jang H.W., Suh S., Min Y.K., Chung J.H. (2013). Associations between body mass index and clinico-pathological characteristics of papillary thyroid cancer. Clin. Endocrinol..

[52] Xu L., Port M., Landi S., Gemignani F., Cipollini M., Elisei R., Goudeva L., Müller J.A., Nerlich K., Pellegrini G. (2014). Obesity and the risk of papillary thyroid cancer: A pooled analysis of three case-control studies. Thyroid.

[53] Nagarajah J., Le M., Knauf J.A., Ferrandino G., Montero-Conde C., Pillarsetty N., Bolaender A., Irwin C., Krishnamoorthy G.P., Saqcena M. (2016). Sustained ERK inhibition maximizes responses of BrafV600E thyroid cancers to radioiodine. J. Clin. Investig..

[54] Whale A.S., Huggett J.F., Cowen S., Speirs V., Shaw J., Ellison S., Foy C.A., Scott D.J. (2012). Comparison of microfluidic digital PCR and conventional quantitative PCR for measuring copy number variation. Nucleic Acids Res..

[55] Day E., Dear P.H., McCaughan F. (2013). Digital PCR strategies in the development and analysis of molecular biomarkers for personalized medicine. Methods.

[56] Huggett J.F., Cowen S., Foy C.A. (2015). Considerations for digital PCR as an accurate molecular diagnostic tool. Clin. Chem..

[57] Torres-Adorno A.M., Vitrac H., Qi Y., Tan L., Levental K.R., Fan Y.Y., Yang P., Chapkin R.S., Eckhardt B.L., Ueno N.T. (2019). Eicosapentaenoic acid in combination with EPHA2 inhibition shows efficacy in preclinical models of triple-negative breast cancer by disrupting cellular cholesterol efflux. Oncogene.

[58] Lee J.Y., Park J.H., Choi H.J., Won H.Y., Joo H.S., Shin D.H., Park M.K., Han B., Kim K.P., Lee T.J. (2017). LSD1 demethylates HIF1α to inhibit hydroxylation and ubiquitin-mediated degradation in tumor angiogenesis. Oncogene.

