# Correction: Park et al. *ABCA1*-Mediated EMT Promotes Papillary Thyroid Cancer Malignancy through the ERK/Fra-1/ZEB1 Pathway. *Cells* 2023, *12*, 274

**DOI:** 10.3390/cells13221821

**Published:** 2024-11-05

**Authors:** Ji-Hye Park, Jae-Kyung Myung, Sun-Joo Lee, Hyewon Kim, Soyeon Kim, Seung-Bum Lee, Hyosun Jang, Won-Il Jang, Sunhoo Park, Hyunwon Yang, Sehwan Shim, Min-Jung Kim

**Affiliations:** 1 Laboratory of Radiation Exposure & Therapeutics, National Radiation Emergency Medical Center, Korea Institute of Radiological & Medical Science, Seoul 01812, Republic of Korea; 2 OPTOLANE Technologies Inc., Seongnam 13494, Republic of Korea; 3 Department of Pathology, College of Medicine, Hanyang University, Seoul 01812, Republic of Korea; 4 Laboratory of Experimental Pathology, Departments of Pathology, Korea Institute of Radiological & Medical Science, Seoul 01812, Republic of Korea; 5 Biohealth Convergence, Seoul Women’s University, Seoul 01812, Republic of Korea

**Error in Figure**

In the original publication [1], there was a mistake in Figure 6A. The images of the expression of both ABCA1 and Fra-1 on Metastasis PTC in Figure 6A in the original manuscript were duplicates. 

We presume that the duplicate photos may have been included due to an error in the process of taking representative photos of the tissue slides from multiple patients, and the identical photos may have been attached during the process of editing the photos to construct figures. The corrected Figure 6A appears below. 

The authors state that the scientific conclusions are unaffected. This correction was approved by the Academic Editor. The original publication has also been updated.

## Figures and Tables

**Figure 6:**
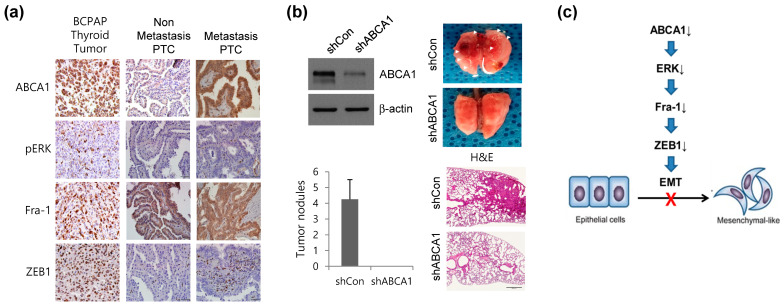
ABCA1 is associated with lung metastasis. (**a**) Representative images indicating protein-stained tumor tissues from mice injected with BCPAP cells and patients with thyroid cancer. Scale bar: 100 μm. (**b**) Cell lysates from BCPAP cells stably expressing control short hairpin RNA (shRNA: shCon) or ABCA1 shRNA (shABCA1) were analyzed via immunoblotting (upper). A representative image of histological analysis of the lungs isolated from mice injected with shCon in the tail vein or ABCA1-knocked-down (shABCA1) BCPAP cells (left). Arrowheads and hematoxylin and eosin (H&E) staining images indicate lung metastatic nodules (right). Data were quantified by counting the number of surface lung nodules (upper left). Error bars indicate the means ± S.E.M. * *p* < 0.05 versus shCon (Student’s *t*-test). (**c**) Proposed model for the regulation of EMT by ABCA1. In the absence of ABCA1, decreased expression of Fra-1 is due to reduced ERK activity, which leads to decreased Fra-1 binding to the promoter region of *ZEB1* EMT-TF. This reduces the expression of ZEB1, and the reduced expression of ZEB1 suppresses EMT.
