# Supplementary material for: ABCA1-Mediated EMT Promotes Papillary Thyroid Cancer Malignancy through the ERK/Fra-1/ZEB1 Pathway
Source: Cells. 2023 Jan 10;12(2):274. doi: 10.3390/cells12020274 (PMC9857273; doi:10.3390/cells12020274)
Supplement: Supplementary file 1 [file cells-12-00274-s001.zip › cells-2115119-supplementary.pdf]

ABCA1 promotes the malignancy of papillary thyroid cancer through ERK/Fra-1/ZEB1 signaling

Ji-Hye Park[a,d,1], Jae Kyung Myung[a,c,1], Sun-Joo Lee[b], Hyewon Kim[b], Soyeon Kim[a], Seung Bum Lee[a], Hyosun Jang[a,] Won Il Jang[a,b], Sunhoo Park[a,b], Hyunwon Yang[e], Sehwan Shim[a,*], Min-Jung Kim[a,*]

Supplementary material and method

Microarray analysis

Microarray analysis was performed using Affymetrix Whole Transcript Expression Arrays according to the manufacturer's protocol (GeneChip WT Pico Reagent Kit, Affymetrix Inc., Santa Clara, CA, USA). cDNA was synthesized using the GeneChip WT Pico Amplification Kit (Affymetrix) according to the manufacturer's instruction. Subsequently, the sense cDNA was fragmented and biotin-labeled with terminal deoxynucleotidyl transferase (TdT) using the GeneChip WT Terminal labeling kit (Affymetrix). Approximately 5.5 μg of labeled DNA target was hybridized to the Affymetrix GeneChip Human 2.0 ST Array at 45 °C for 16 h. Hybridized arrays were washed and stained on a GeneChip Fluidics Station 450 and scanned on a GCS3000 scanner (Affymetrix). Signal values were computed using Affymetrix GeneChip Command Console software. The data were summarized and normalized using the robust multi-average (RMA) method implemented in Affymetrix Power Tools (APT). We exported the result with gene level RMA analysis and performed differentially expressed gene (DEG) analysis. The statistical significance of the expression data was determined using fold change.

Western blot analysis

Cells were lysed in a buffer [40 mM Tris-HCl (pH 8.0), 120 mM NaCl, 0.1% Nonidet-P40] supplemented with protease and phosphatase inhibitors, and the lysates were subjected to western blot analysis as described in a previous study [1]. Antibodies specific for the following factors were used for western blotting: ABCA1 (Novus Biologicals, Abingdon, UK); EGFR, Fra-1, Slug, and β-actin (Santa Cruz Biotechnology, Santa Cruz, CA, USA); vimentin, Snail, Twist (Abcam, Cambridge, MA, USA), phospho-EGFR, phospho-Src, Src, phospho-PI3K,

PI3K, phospho-AKT, AKT, phospho-MEK, MEK, ERK, phospho-JNK, JNK, phospho-P38, p38 (Cell Signaling Technology, Danvers, MA, USA), N-cadherin (BD Bioscience, Franklin Lakes, NJ, USA); and ZEB1 (Sigma).

RT–PCR and qRT–PCR

Total RNA from formalin-fixed paraffin-embedded (FFPE) TC tissues was isolated using an RNeasy FFPE kit (QIAGEN, Hilden, Germany), and thyroid cells were isolated using TRIzol reagent (Invitrogen) according to the manufacturer's instructions. Reverse transcription–PCR (RT-PCR) was performed according to the instructions provided with the ReverTra Ace qPCR RT Kit (TOYOBO, Osaka, Japan) and AccuPower RocketScript RT-qPCR PreMix (Bioneer, Daejeon, Korea). To quantify RNA expression levels, qRT-PCR was performed on the LightCycler 96 Real-Time PCR System (Roche, Mannheim, Germany) using SYBR Green dye (Applied Biosystems, Foster City, CA, USA) as described by the manufacturer, and data were normalized to the expression of the control gene *GAPDH*. The specific primers used are given in Supplementary Table 3.

Lentiviral overexpression and knockdown system

To establish luciferase-overexpressing cell lines for Luc imaging, Luc cDNA was subcloned into a lentiviral pLVX-puro vector (Clontech). To generate ABCA1-knockdown stable cell lines using short hairpin RNA (shRNA), two different pLKO.1–shABCA1 lentiviral vectors (shABCA1 #1 and #2) were purchased from Sigma–Aldrich (MISSION). 293T cells were transfected with each lentiviral vector and packaging DNA containing pMD2g and psPAX by using Lipofectamine 2000 (Invitrogen). After transfection for 48 h, the medium containing the viruses was harvested, filtered, and transferred to the target cells along with 8 μg ml^{-1} polybrene (Sigma, St. Louis, MO).

Immunocytochemistry

Cells were seeded at a density of 3×10^4 cells/well in a 6-well plate and washed with PBS. After fixation with 4% PFA and permeabilization with 0.1% Triton X-100 in PBS, the cells

were incubated at 4 °C overnight with anti-ABCA1, vimentin, ZEB1, or Fra-1 primary antibody in PBS with 1% bovine serum albumin and 0.1% Triton X-100. For the visualization of stained proteins, the cells were further incubated with secondary antibody against anti-mouse immunoglobulin/FITC or anti-rabbit immunoglobulin/PE (1:400, BD Bioscience) for 1 h. The nuclei were counterstained with DAPI (Sigma). A fluorescence microscope (Olympus) was used for immunofluorescence detection.

Immunohistochemistry

Tissue sections from paraffin-embedded blocks of xenograft tumors and patients with PTC were stained with hematoxylin and eosin (H&E) to define the most morphologically representative and non-necrotic areas. Immunohistochemical staining was performed using an automated immunostainer (Lab Vision Auto Stainer 480S, Fremont, CA, USA), following the manufacturer's instructions. After deparaffinization, the slides were boiled in a pressure cooker for antigen retrieval and immunostained with antibodies targeting ABCA1, pERK, Fra-1, and ZEB1. Slides were sequentially incubated with HRP polymer (Thermo Fisher Scientific) and DAB chromogen substrate. The DAB-treated slides were stained with Mayer's hematoxylin and mounted using Richard-Allan Scientific Mounting Medium (Thermo Fisher Scientific).

ChIP–qPCR assays

ChIP assays were performed as described in a previous study [2]. Briefly, the crosslinked cell pellets were resuspended in lysis buffer, sonicated, and centrifuged. Subsequently, the cell lysates were immunoprecipitated with anti-Fra-1 for overnight at 4 °C. The precipitates were washed and eluted, following which, they were reverse-crosslinked with 20 μL 5 M NaCl by incubating at 65 °C overnight. The DNA fragments were precipitated from the eluate and dissolved in ddH_2O. For quantification of the ChIP analysis, we performed real-time qPCR as described above using the specific primers given in Supplementary Table 4.

Mouse metastasis model and histopathological analysis

Female BALB/c nude mice (6–8-week-old) were purchased from the Orient Bio Company

(Seoul, South Korea). BCPAP/Luc cells were stably transfected with a lentiviral luciferase vector, and 1 × 10^6 cells suspended in 100 μL of normal saline were injected into the tail vein of the mice. Control mice were injected with 100 μL of normal saline. Each group comprised four animals. Every week, firefly luciferase bioluminescence signals of Luc stable cells were analyzed using the In-vivo Xtreme Imaging System (Bruker, Billerica, MA, USA). Following bioluminescence analysis, the animals were sacrificed, and lung metastatic colonization was monitored and quantified using histological analysis.

Table S1. siRNA sequences used in this study.

siRNA	Sequence
ABCA1 siRNA #1	GGUAUGAGGACAACAACUAUU
ABCA1 siRNA #2	GCUGAAACCUUCUAUGACAUU
Fra-1 siRNA #1	CACCAUGAGUGGCAGUCAGdTdT
Fra-1 siRNA #2	CUGACUGCCACUCAUGGUGdTdT
ZEB1 siRNA #1	GGUAGAUGGUAAUGUAAUAUU
ZEB1 siRNA #2	CCUAGUCAGCCACCUUUAAUU
ERK siRNA #1	GACCGGAUGUUAACCUUUAUU
ERK siRNA #2	CACCAACCAUCGAGCAAAUUU

Table S2. Primers and probes of Dr.PCR used in this study.

Primer and probe name	Sequence
ABCA1 forward	TGCAAGGCTACCAGTTACATT
ABCA1 reverse	TTAGTGTTCTCAGGATTGGCT
ABCA1 probe	FAM-CCTACCAAGGGAGAAACTGGCTGC-BHQ1-3
GAPDH forward	CCCAGCAAGAGCACAAGAGGAAG
GAPDH reverse	GAGGGGAGATTCAGTGTGGTGGG
GAPDH probe	SFC620-AGT/iFAM_T/-ACCCTCACTGCTGGGGAGTCCCTGC-SFCQ2

Table S3. Primers of qRT-PCR used in this study.

Primer name	Sequence
ABCA1 forward	TGCAAGGCTACCAGTTACATT
ABCA1 reverse	TTAGTGTTCTCAGGATTGGCT
N-cadherin forward	ATCCGGTCCGATCTGCAGCC
N-cadherin reverse	GTGGCCCCCAGTCGTTCAGGTA
Vimentin forward	AAACTTAGGGGCGCTCTTGT
Vimentin reverse	GAGGGCTCCTAGCGGTTTAG
Snail forward	CGAGTGGTTCTTCTGCGCTA
Snail reverse	GGGCTGCTGGAAGGTAAACT
Slug forward	GCTACCCAATGGCCTCTCTC
Slug reverse	CTTCAATGGCATGGGGGTCT
ZEB1 forward	ATGACCTGCCAACAGACCAG
ZEB1 reverse	CCTCCCAGCAGTTCTTAGCA
Twist forward	GGAGTCCGCAGTCTTACGAG
Twist reverse	TCTGGAGGACCTGGTAGAGG
Fra-1 forward	CCGGGCATGTTCCGAGACTT
Fra-1 reverse	ACTCATGGTGTTGATGCTTGGCAC
GAPDH forward	CGAGATCCCTCCAAAATCAA
GAPDH reverse	TGTGGTCATGAGTCCTTCCA

Table S4. Primers of ChIP-qPCR used in this study.

Primer name		Sequence
ZEB1 promoter (1)	forward	TCCTGTCTAGAAGCAGATACGAAG
	reverse	CAGACGTCTTTAAAATGCAAGTG
ZEB1 promoter (2)	forward	AACAGATGACTTAAGGGGGG
	reverse	CTGCAGCGATCAAGAACCTC

1. Park, J.H.; Kim, Y.H.; Park, E.H.; Lee, S.J.; Kim, H.; Kim, A.; Lee, S.B.; Shim, S.; Jang, H.; Myung, J.K.; et al. Effects of metformin and phenformin on apoptosis and epithelial-mesenchymal transition in chemoresistant rectal cancer. *Cancer Sci.* **2019**, *110*, 2834–2845. DOI:10.1111/cas.14124.
2. Lee, J.Y.; Park, J.H.; Choi, H.J.; Won, H.Y.; Joo, H.S.; Shin, D.H.; Park, M.K.; Han, B.; Kim, K.P.; Lee, T.J.; et al. LSD1 demethylates HIF1α to inhibit hydroxylation and ubiquitin-mediated degradation in tumor angiogenesis. *Oncogene*. **2017**, *36*, 5512–5521. DOI:10.1038/onc.2017.158.
